# Prevalence of Anaemia among Pregnant Women at Booking in the University of Uyo Teaching Hospital, Uyo, Nigeria

**DOI:** 10.1155/2014/849080

**Published:** 2014-05-19

**Authors:** Olujimi A. Olatunbosun, Aniekan M. Abasiattai, Emem A. Bassey, Robert S. James, Godwin Ibanga, Anyiekere Morgan

**Affiliations:** ^1^Department of Obstetrics/Gynaecology, University of Uyo Teaching Hospital, PMB 1136, Uyo 520001, Nigeria; ^2^Department of Community Health, University of Uyo Teaching Hospital, Uyo 520001, Nigeria

## Abstract

*Background*. Anaemia with an estimated prevalence of 35–75% among pregnant women is a major cause of maternal deaths in Nigeria. * Objective*. To determine the prevalence of anaemia, associated sociodemographic factors and red cell morphological pattern among pregnant women during booking at the University Teaching Hospital, Uyo. * Material and Methods*. A cross-sectional analytical study of 400 women at the booking clinic over a 16-week period. The packed cell volume and red cell morphology of each pregnant woman were determined. Their biodata, obstetric and medical histories, and results of other routine investigations were obtained with questionnaires and analyzed with SPSS Package version 17.0. *Results*. The mean packed cell volume was 31.8% ±3.2 and 54.5% of the women were anaemic. The commonest blood picture was microcytic hypochromia and normocytic hypochromia suggesting iron deficiency anaemia. Anaemia was significantly and independently related to a history of fever in the index pregnancy (OR = 0.4; *P* = 0.00; 95% CI = 0.3–0.7), HIV positive status (OR = 0.2; *P* = 0.01; 95% CI = 0.1–0.6), and low social class (OR = 0.3; *P* = 0.00; 95% CI = 0.2–0.7). *Conclusion*. Women need to be economically empowered and every pregnant woman should be encouraged to obtain antenatal care, where haematinics supplementation can be given and appropriate investigations and treatment of causes of fever and management of HIV can be instituted.

## 1. Introduction

The importance of good haemoglobin concentration during pregnancy for both the woman and the growing foetus cannot be overemphasized. Being a driving force for oxygen for the mother and foetus, a reduction below acceptable levels can be detrimental to both [1]. Traditionally, anaemia is defined as a decrease in the ability of blood to carry oxygen due to a decrease in the total number of erythrocytes (each having a normal quantity of haemoglobin), a diminished concentration of haemoglobin per erythrocyte, or a combination of both [2]. A haemoglobin concentration below 11.0gldl or packed cell volume (PCV) of less than 33.0% is regarded as anaemia during pregnancy by the World Health Organization (WHO) [3].

It is one of the most intractable public health problems in developing countries and the commonest complication in pregnancy in sub-Saharan Africa, more so with the advent of the HIV/AIDS pandemic [4]. The WHO estimates that anaemia affects over half of the pregnant women in developing countries [5]. Recent estimates in the developing countries including Nigeria put the prevalence at 60.0% in pregnancy and about 7.0% of the women are said to be severely anaemic [1, 5, 6]. The high prevalence and the aetiological factors responsible for anaemia in pregnancy are multiple and their relative contributions are said to vary by geographical area and by season [7].

Anaemia in pregnancy may be relative or absolute [8]. Relative anaemia is a normal physiological phenomenon that occurs in pregnancy due to larger increase in plasma volume (approximately 45.0% in singleton and 50.0–60.0% in twin gestation) than in red cell mass, resulting in the well-known physiological anaemia of pregnancy. Absolute anaemia involves a true decrease in red cell mass, involving increased red cell destruction as in haemoglobinopathy, malaria, and bacterial infection like urinary tract infection; increased red cell loss as in bleeding; or decreased red cell production as in nutritional deficiency or chronic disease [8, 9]. Predisposing factors include young age, grand multiparity, low socioeconomic status, illiteracy, ignorance, and short interpregnancy intervals [10]. Infection with hookworm and intestinal helminthes causes gastrointestinal blood loss resulting in depletion of the iron stores and consequently impaired erythropoiesis. They also lead to mal-absorption and inhibition of appetite, thereby worsening micronutrients deficiency and maternal anaemia.

The principle of anaemia prevention in the study centre is control of malaria and haematinics supplementation [3]. All pregnant women receive routine daily supplementation of elemental iron and folic acid. Protection against malaria is usually achieved through the use of insecticide treated bed nets, intermittent preventive treatment of asymptomatic pregnant women, and early diagnosis and prompt and effective case management of malaria. Other interventions include HIV screening and management, health education on diet, cooking, and early diagnosis and treatment of anaemia which depends on the severity and its cause as well as the gestational age of the patient. Correction of anaemia in pregnancy can be achieved either with haematinics or by blood transfusion.

A key component of a safe motherhood initiative is to reduce maternal mortality by half through the eradication of anaemia during pregnancy [11]. The management of anaemia in pregnancy is a potentially feasible and cost-effective intervention to reduce maternal, foetal, and perinatal mortality and morbidity. However, the actual prevalence rates of pregnancy related conditions for many individual countries and communities are not known. Thus, it was recommended at the African regional consultation on the control of anaemia in pregnancy of the WHO that simple studies of prevalence and aetiology should be undertaken [11, 12].

Many of the predisposing factors to anaemia in pregnancy are controllable and may lead to women becoming pregnant with anaemia; thus there is need for basic prevalence statistics to create awareness on the magnitude of anaemia in pregnancy in our environment and also to formulate strategies to reduce its adverse health consequences in order to improve maternal health and reduce poor perinatal outcome. Information on the prevalence would also be useful for the managers of health institutions and for district, provincial, and national maternal, child, and women's health programme development [11, 12]. Hence, this study aims to determine the prevalence of anaemia and the red cell morphological pattern among pregnant women at the booking clinic of the University of Uyo Teaching Hospital (UUTH), Uyo. The results from this study will reveal the magnitude of this problem in our environment and also provide relevant data to strengthen planning on the prevention of anaemia in pregnancy, thus helping to reduce the prevalence of anaemia in pregnancy and the morbidity and mortality associated with it.

## 2. Materials and Methods

### 2.1. Study Area

This cross-sectional study was carried out in the antenatal clinic of the University of Uyo Teaching Hospital. This is the only tertiary health facility in Akwa Ibom State and is situated in the South-South geopolitical zone of Nigeria. Uyo, the capital city, is located in the rain forest belt of Nigeria with an annual rainfall of 2430.0 mm. However, there is a dry season from December to February when monthly rainfall is low. The teaching hospital serves as a training institution for undergraduates and also for resident postgraduate doctors. It also serves as a major referral centre for both government-owned and privately owned hospitals in Uyo and its environs.

### 2.2. Estimation of the Sample Size

Average prevalence of anaemia in pregnancy in developing countries including Nigeria is put at 60.0% [7, 8]. The estimate from this study was desired to be within five percent of the actual prevalence with 95 percent confidence level. The sample size was calculated using the Kish Leslie formula for cross-sectional studies [3]:

(1) $$\begin{array}{c} n=\frac{Z^{2}Pq}{d^{2}}, \end{array}$$

where *n* is the desired sample size and *Z* is the standard normal deviate usually set at 1.96, which corresponds to the 95% confidence interval. *P* is the proportion of pregnant women with anaemia, which is 60.0%. *q* is complementary proportion equivalent to one (1) minus *P*; that is, 1 − 0.6 equal to 0.4. *d* is the degree of accuracy desired (absolute precision), which is 5.0% (0.05).

Thus

(2) $$\begin{array}{c} n=\frac{1.062\times 0.6\left(1-0.6\right)}{0.05^{2}}=\frac{0.921984}{0.0025}=369. \end{array}$$

Hence about 369 subjects were needed for the study. Since followup was not needed in the study, attrition rate was not necessary. However, there was a deliberate increase in sampling size to 400.

### 2.3. Patients' Selection

A total of 3900 women booked for antenatal care (ANC) annually from review of the previous year record, resulting in an average booking rate of 75 women per week. The study was designed to be performed over 16 weeks during which an estimated 1200 women were expected to book for antenatal care. The attendance list of the women at each booking clinic served as the sampling frame. The attendance number of each woman at every booking clinic was written on separate slips of paper. These were then thoroughly mixed in a container from where the first woman was picked randomly by balloting. If the woman did not meet the inclusion criteria, a new number was drawn until one met the criteria. The remaining numbers of women were selected through systematic sampling, at fixed intervals (sampling interval: 1200/400 = 3) of every third number on the sampling frame to make up to the required 25 women per week. The inclusion criteria included pregnant women at their first antenatal visit who were willing to participate in the study. Those excluded were pregnant women at their follow-up antenatal visit and those who had received blood transfusions in the index pregnancy or were already receiving treatment for anaemia in pregnancy before their booking visit.

### 2.4. Data Collection

Data were collected over a period of sixteen weeks (July–October, 2012); during that time 400 pregnant women were recruited at their first antenatal visit. The women were interviewed with copies of a structured questionnaire by trained registrars in the Department of Obstetrics and Gynaecology to ensure as much as possible that the necessary information was obtained. The following information was recorded: maternal age, parity, gestational age, last child birth, last menstrual period, level of education and occupation of the women and their husband, history of fever in the index pregnancy, presence of any chronic illnesses, and history of vaginal bleeding in present pregnancy.

Each woman in the study was allotted to one of the five social classes based on her level of education and her husband's occupation according to a scoring system designed by Olusanya et al. [13]. The social class for single or separated women was based on their occupation and their educational status. Social classes 1 and 2, the upper class, represented the elites, the professionals like the doctors, lawyers, bankers, those in managerial position, and so forth. Class 3 represented the middle class of nurses, clerks, technicians, artisans, and so forth, while classes 4 and 5 represented the lowest rung on the socioeconomic ladder.

Packed cell volume and red cell morphology were done for the women at the time of recruitment. From each of the recruited woman, 5 mls of venous blood was collected from the antecubital vein using plastic disposable syringes into sample bottles containing ethylene diamine-tetra acetic acid (EDTA).

### 2.5. Packed Cell Volume Estimation

Two capillary tubes labelled for each subject were filled with blood to about 2/3 of the length of each tube and one end of each of these tubes was then sealed with plasticin. This was to ensure that the average of the two values obtained was used for calculation. Several labelled samples were assembled in the centrifuge (haematocrit machine) and spun at 5000 revolutions per minute for 5 minutes. When the machine rotated to a halt, the cover was opened, the capillary tubes removed, and the packed cell volume was read from a Hawksley microhaematocrit reader.

### 2.6. Blood Film Microscopy for Red Cell Morphology

A small drop of blood was placed in the centre of a labelled glass slide about 1-2 cm from one end. Another smooth glass slide was then used as spreader at an angle of 450 to the slide. The blood film was air dried and then flooded with Leishman's stain. After 2 minutes, water was added and the film stained for 5–7 minutes. It was then washed in a stream of buffered water until it acquired a pinkish tinge. The back of the slide was then wiped clean and the slide set upright, allowed to dry and then examined under the microscope. Microcytic hypochromic morphology was considered consistent with iron deficiency, while a macrocytic blood picture was considered megaloblastic anaemia. A combination of the two was interpreted as dimorphic anaemia.

The results of the other routine investigations were retrieved from the laboratory and entered into the questionnaires. The laboratory investigations were carried out at the haematology laboratory of the teaching hospital. Women that were diagnosed to be anaemic (packed cell volume of less than 33%) were counselled on the need for further investigations and were referred to their obstetricians for further management. All the women were given a prescription of haematinics.

Ethical approval was obtained from the ethical committee of the University of Uyo Teaching Hospital. The details of the study were thoroughly explained to all women booking for antenatal care at the beginning of the clinic. An informed consent was obtained in writing before recruiting each subject into the study. The participants retained the absolute right and freedom to decline from participating or withdrawing from the study at any time with no consequence to them. They were assured that opting out would not compromise the quality of care they receive from the antenatal clinic. The study was carried out under strict confidentiality as participants and their samples were identified by initials and serial numbers on their questionnaires, laboratory forms, and specimen bottles. Those that participated in the study had the benefit of early diagnosis of anaemia when present. This allowed for early treatment and ultimate prevention of the consequences of anaemia in the index pregnancy.

### 2.7. Data Analysis

Data were analysed using the statistical package for social sciences (SPSS package) version 17.0. Descriptive statistics were computed for all relevant variables. Comparative analysis was done with the chi-square test and level of significance was set at *P* < 0.05. Association between anaemia and some risk factors in pregnancy was tested using chi-square and multivariate analysis of risk factors was done.

## 3. Results

Four hundred pregnant women were recruited over a period of 16 weeks and all of them consented and accepted to participate in the study. The mean age of the women was 28.9 ± 4.5 with a range of 17 to 45 years. The mean parity was 1.0 ± 1.1 with a parity of 0 to 4. The mean packed cell volume (PCV) was 31.8% ± 3.3 with a range of 20 to 42%. The mean gestational age at booking was 23.3 weeks ± 7.2. Among the pregnant women with previous delivery experience, the mean interpregnancy interval was 2.1 ± 0.9 years with a range of 1.0 to 6.5 years.

Two hundred and eighteen women (54.5%) were anaemic, majority (61.0%) of the anaemic women had mild anaemia, and 38.5% had moderate anaemia, while 0.5% had severe anaemia. One hundred and seventy-nine women (44.8%) had normocytosis, 33.0% had microcytosis, and 80.5% had hypochromic red cells.

Table 1 shows the sociodemographic characteristics of the women. More than half (57.0%) of the women had acquired tertiary level education, while 61.5% of them were in the middle and lower social classes.

Table 2 shows the clinical characteristics of the women. Majority of the women (49.0%) booked for antenatal care in the second trimester, while 13.8% of the women were of uncertain gestational age. A total of 45.3% of the women had experienced fever in the course of the index pregnancy. Twenty-six (6.5%) of the women were HIV seropositive, while 14.8% had the sickle cell trait.

Majority of the women with normal red cell morphology (normocytosis and normochromia) were not anaemic (62.0% and 71.8%, resp.); however, 38.0% of them had anaemia. Also, majority (66.5%) of those with microcytic hypochromic red cell morphology were anaemic (Table 3).

There was an inverse relationship between the prevalence of anaemia and the level of education of the women (*x* ^2^ = 17.6, *P* = 0.00). The proportion of anaemic women increased with decrease in the level of education. Anaemia was significantly more common in women of lower social class (*x* ^2^ = 14.5, *P* = 0.00) (Table 4).

On univariate analysis, there was significant association between history of fever in the index pregnancy, vaginal bleeding in the index pregnancy, and HIV status of the women with anaemia. However, there was no significant association between the gestational age (trimester) at booking and anaemia (*x* ^2^ = 1.9, *P* = 0.58) (Table 5).

However, multivariate analysis showed that anaemia was significantly and independently related to history of fever in index pregnancy (OR = 0.4; *P* = 0.00; 95% CI = 0.3–0.7), HIV positive status (OR = 0.2; *P* = 0.01; 95% CI = 0.1–0.6), and low social class (OR = 0.3; *P* = 0.00; 95% CI = 0.2–0.7) as shown in Table 6.

## 4. Discussion

The diagnosis of anaemia during booking among pregnant women is essential as it affords one the opportunity to institute interventions to prevent the complication of anaemia especially considering the prevalent high maternal and perinatal morbidity and mortality associated with anaemia in pregnancy in the tropics [1]. Data from the literature in developing countries have reported prevalence of anaemia in pregnancy that ranged from 35.0 to 75.0% [5]. The prevalence of anaemia in this study was 54.5% which is in the middle range when compared to findings from other studies in Nigeria [1, 3, 4, 6, 8, 14, 15] and from countries in South Eastern Africa [7, 12].

The high prevalence of anaemia in this study is probably related to the low socioeconomic status of the women, which may have impact on their nutritional status and health seeking behavior [3, 8]. The prevalence of anaemia in this study is slightly lower than the 56.0% quoted by WHO for prevalence of anaemia in Africa based on the 1988 data [6] implying that even after 25 years, the situation has only marginally improved.

Most of the women in this study had anaemia of mild to moderate severity with only 0.3% being severely anaemic. These findings are similar to the findings from Ugwuja et al. [16] and those of Aluka et al. [17] and Adinma et al. [10] except for the absence of severe anaemia in these other studies.

The mean PCV in this study was 31.8%. By WHO standard, this is anaemia. The number of pregnant women with anaemia decreased with increase in maternal age. These findings agree with the studies done in other parts of Nigeria [6, 14] and other African countries [9, 12].

It is generally believed that anaemia in pregnancy increases with rising parity, due to repeated drain on iron stores [10]. However, this study like those of other researchers [4, 8, 14] has revealed an inverse relationship between parity and anaemia as the percentage of anaemic pregnant women decreased as parity increased. The possible reasons for this finding include increased awareness of the value of drugs and good diet with increasing parity as well as increased interaction with other pregnant women at the clinic [4, 8, 14]. The effect of these would to some extent neutralize those of rising parity. Another possible reason is that women of higher parity booked for ANC earlier in gestation, when iron requirements are still low compared to women of lower parity who booked later in pregnancy when iron requirements are much higher, thus predisposing them to anaemia. The implication of this is that, in a malarial endemic society like ours, the nulliparous/primigravidae who are more susceptible to malaria waited until parasitaemia had adversely affected them before booking [4, 8, 14], thus exposing them to a higher risk of anaemia.

It was interesting to note that despite the high level of education of most of the pregnant women in this study, the majority of them were still in the middle to low social class as also noted by Bukar et al. [8] and Lamina and Sorunmu [18] in their studies. This is because, though majority of the husbands of these women were in the skilled labour class, a significant percentage of them were in the unskilled labour class and only about a quarter were professionals. About 56.3% of the pregnant women were in the unskilled labour class. The low socioeconomic status of the women may have a significant impact on their nutritional status and health seeking behavior. Women with low socioeconomic status tend to consume diets that are low in micronutrients, animal protein, and vitamins but high in carbohydrate and phytates which interfere with intestinal uptake of iron and other trace minerals such as zinc and calcium [19]. This indicates that economic empowerment of women would play a very important role in reducing the prevalence of anaemia in our environment.

The percentage of women with anaemia was lowest among those that booked for antenatal care in the first trimester. This finding is in agreement with findings of Komolafe et al. [6] in Ilesha and Bukar et al. [8] in Gombe. This is probably due to the fact that the majority of our women booked in the second and third trimester of pregnancy. The dilutional effect of pregnancy and increased fetal demand for haematopoietic factors are maximal after the first trimester. Also, underlying maternal diseases and untreated anaemia in early pregnancy are likely to worsen in the course of pregnancy.

Approximately 57.6% of the parous women in this study had an interpregnancy interval of greater than 2 years. The high level of education of the women as well as the fact that a large proportion of them were civil/public servants may contribute to this trend. Similar to the findings of Bukar et al. [8], the percentage of women with anaemia was higher among those with an interpregnancy interval of less than 2 years. The history of febrile illness in the pregnant women was significantly associated with anaemia. Fever may be a proxy for malaria, a major cause of both anaemia and fever especially in a malaria holoendemic area like Nigeria. Among the 26 women who tested positive for HIV 88.5% were anaemic. This is not unexpected as HIV infection is a recognized risk factor for anaemia. Suggested mechanisms include a direct effect of the virus itself, bone marrow suppression due to cytokine release, and anaemia as a result of chronic inflammation or opportunistic infections which may be further exacerbated by antiretroviral drugs like zidovudine, a component of highly active antiretroviral therapy.

Normocytic hypochromia and microcytic hypochromia blood picture, the most common morphological types of anaemia found in this study, are characteristic of iron deficiency anaemia. In developing countries like ours, anaemia in pregnancy is commonly believed to result from nutritional deficiencies especially iron. The gold standard for making a diagnosis of iron deficiency anaemia examination of stained bone marrow aspirate for haemosiderin is invasive. Serum ferritin measurement on the other hand is costly and largely not available in most centers in the country and is elevated in the presence of inflammation which is not uncommon in our environment. A blood picture suggestive of iron deficiency was found to complicate 89.9% of all case of anaemia in this study. This is higher than the 64.0% reported by Dorothy et al. [20]. The high percentage of iron deficiency in this study could be as a result of the large proportion of women of low socioeconomic class. Iron needs increase greatly during the second and third trimesters of pregnancy and that was when 78.0% of women that were certain of their gestational age booked for antenatal care.

Following multivariate analysis, the risk factors in this study were history of fever in index pregnancy, HIV positive status, and low social class. This shows that the interventions to prevent and treat anaemia during the antenatal care visits are appropriate and need to be pursued vigorously. Also investigations into other causes of fever apart from malaria need to be looked into and treated appropriately.

This study was hospital based and as such may not be truly reflective of the situation in the state due to selection bias. Pregnant women utilizing our centre are also more likely to be educated, of higher socioeconomic status than the typical pregnant woman in the community. This study also depended on results obtained from laboratory procedures which may not be error proof and these errors may arise from human factors or from the equipment used for the procedures. Intestinal helminthes were not screened for because the study area being an urban area has good water supply and environmental sanitation. Besides generally, the level of education of the residents is high, and therefore the incidence of this condition is expected to be low. None of the women in this study had sickle cell anaemia. History of fever in the index pregnancy was used as a proxy for malaria, a major cause of both anaemia and fever especially in a malarial holoendemic area like Nigeria.

## 5. Conclusion

This study has revealed that anaemia in pregnancy is still highly prevalent in our environment. The study has also revealed that the most important risk factors for anaemia in pregnancy in this center are socioeconomic status of women, fever, and HIV seropositive status. The commonest red cell blood pictures among the anaemic clients were microcytic hypochromia and normocytic hypochromia, which are indicative of iron deficiency anaemia. Hence, public health campaigns to create awareness about the importance of early booking for antenatal care are recommended. This will provide opportunity for early detection and treatment of anaemia, as well as the timely institution of preventive measures like haematinics and antimalarials. The socioeconomic situation of the womenfolk should be improved by economically empowering them through provision of salary paying jobs by government since majority of these women are educated. Also efforts should be made towards HIV prevention and treatment in the state. Further studies are needed to identify specific aetiological factors. A study of the prevalence and pattern of anaemia in the community is also recommended. This would aid in planning healthcare services, in reducing the attendant morbidity and mortality and also improving the wellbeing of women in the society in general.

## Figures and Tables

**Table 1:** Sociodemographic characteristics of the women at booking *N* = 400.

Sociodemographic characteristics	Frequency (*n*)	Percent (%)
Age (years)		
<20	3	0.8
20–24	57	14.3
25–29	165	41.3
30–34	129	32.3
35–39	42	10.5
≥40	4	1.0
Marital status		
Single	6	1.5
Married	393	98.3
Separated	1	0.3
Parity		
0	215	53.8
1	93	23.3
2	49	12.3
3	35	8.8
4	8	2.0
Educational level		
Primary/no formal	23	5.8
Secondary	149	37.3
Tertiary	228	57.0
Social class		
1	56	14.0
2	98	24.5
3	119	29.8
4	107	26.8
5	20	5.0
Occupation		
Professional	23	5.8
Skilled labour	152	38.0
Unskilled labour	225	56.3
Husband's occupation		
Professional	61	15.5
Skilled labour	195	49.6
Unskilled labour	137	34.9

**Table 2:** Clinical characteristics of the women *N* = 400.

Clinical characteristics	Frequency	Percent
Trimester		
First	33	8.3
Second	196	49.0
Third	116	29.0
Unknown	55	13.8
Interpregnancy interval		
<2 years	50	42.4
≥2 years	68	57.6
Miscarriage/ectopic		
Yes	186	46.5
No	214	53.5
Bleeding previous pregnancy		
Yes	14	4.2
No	318	95.8
Bleeding index pregnancy		
Yes	34	8.5
No	366	91.5
Fever		
Yes	181	45.3
No	219	54.8
Genotype		
AA	341	85.3
AS	59	14.8
HIV		
Yes	26	6.5
No	374	93.5

**Table 3:** Association between the red cell morphological pattern of the women and anaemia.

RBC morphology	Anaemic (%)	Nonanaemic (%)	*P* value	*χ* ^2^
Normocytosis			0.00	34.5
Yes	68 (38.0)	111 (62.0)		
No	149 (67.4)	72 (32.6)		
Normochromia			0.00	26.5
Yes	22 (28.2)	56 (71.8)		
No	195 (60.6)	127 (39.4)		
Hypochromia			0.00	26.5
Yes	195 (60.6)	127 (39.4)		
No	22 (28.2)	56 (71.8)		
Microcytosis			0.00	33.5
Yes	107 (66.5)	54 (33.5)		
No	110 (46.0)	129 (54.0)		
Macrocytosis			0.00	8.0
Yes	60 (67.4)	29 (32.6)		
No	157 (50.5)	154 (49.5)		

**Table 4:** Association between sociodemographic characteristics of pregnant women at booking and anaemia in the study population.

Characteristics	Anaemic (*n*)	Nonanaemic (*n*)	*χ* ^2^	*P* value
Age (years)			2.6	0.77
<20	2	1		
20–24	35	22		
25–29	91	74		
30–34	64	65		
35–39	23	19		
≥40	2	2		
Marital Status			2.9	0.23
Single	5	1		
Married	211	182		
Separated	1	0		
Parity			9.2	0.06
0	96	72		
1	56	60		
2	38	26		
3	17	23		
4	10	2		
Education level			17.6	0.00
Primary/no formal	11 (47.8)	12 (52.2)		
Secondary	101 (67.8)	48 (32.2)		
Tertiary	105 (46.0)	123 (54.0)		
Social class			14.5	0.00
1	22 (40.0)	33 (60.0)		
2	51 (52.0)	47 (47.8)		
3	60 (50.4)	59 (49.6)		
4	73 (68.2)	34 (31.8)		
5	6 (42.9)	8 (57.1)		

**Table 5:** Association between some clinical characteristics of the women and anaemia.

Clinical characteristics	Anaemic (%)	Nonanaemic (%)	*χ* ^2^	*P* value
GA (trimester)			1.9	0.58
First	15 (45.5)	18 (54.5)		
Second	108 (55.1)	88 (44.9)		
Third	61 (52.6)	55 (47.4)		
Unknown	33 (60.0)	22 (40.0)		
Interpregnancy interval			24.1	0.19
<2 years	32 (64.0)	18 (36.0)		
≥2 years	33 (48.5)	35 (51.5)		
Fever			17.9	0.00
Yes	121 (66.8)	60 (33.2)		
No	96 (43.8)	123 (56.2)		
Bleeding index pregnancy			5.6	0.02
Yes	25 (73.5)	9 (26.5)		
No	192 (52.5)	174 (47.5)		
Genotype			3.4	0.33
AA	179 (52.5)	162 (47.5)		
AS	38 (64.4)	21 (35.6)		
HIV			13.1	0.00
Yes	23 (88.5)	3 (11.5)		
No	194 (51.9)	180 (48.1)		

**Table 6:** Multivariate analysis of risk factors associated with anaemia.

Risk factors	Odds ratio	*P* value	95% confidence interval
Bleeding in index pregnancy	0.5	0.09	0.2–1.1
Fever in index pregnancy	0.4	0.00*	0.3–0.7
HIV positive status	0.2	0.01*	0.1–0.6
Para 1	1.4	0.21	0.8–2.3
Para 2	1.1	0.69	0.6–2.1
Para 3	2.1	0.05	1.0–4.5
Para 4	0.3	0.10	0.1–1.3
Social class 2	0.6	0.15	0.3–1.2
Social class 3	0.3	0.00*	0.2–0.7

*Statistically significant *P* values.
